# Fine Particulate Matter National Ambient Air Quality Standards: Public Health Impact on Populations in the Northeastern United States

**DOI:** 10.1289/ehp.7822

**Published:** 2005-05-10

**Authors:** Philip R.S. Johnson, John J. Graham

**Affiliations:** Northeast States for Coordinated Air Use Management (NESCAUM), Boston, Massachusetts, USA

**Keywords:** air pollution, National Ambient Air Quality Standards, northeastern United States, particulate matter, PM_2.5_, populations, public health, sensitive, susceptible

## Abstract

In this article we identify the magnitude of general and susceptible populations within the northeastern United States that would benefit from compliance with alternative U.S. Environmental Protection Agency (EPA) annual and 24-hr mass-based standards for particulate matter (PM) with an aerodynamic diameter ≤2.5 μm (PM_2.5_). Understanding the scale of susceptibility in relation to the stringency or protectiveness of PM standards is important to achieving the public health protection required by the Clean Air Act of 1970. Evaluative tools are therefore necessary to place into regulatory context available health and monitoring data appropriate to the current review of the PM National Ambient Air Quality Standards (NAAQS). Within the New England, New Jersey, and New York study area, 38% of the total population are < 18 or ≥65 years of age, 4–18% of adults have cardiopulmonary or diabetes health conditions, 12–15% of children have respiratory allergies or lifetime asthma, and 72% of all persons (across child, adult, and elderly age groups) live in densely populated urban areas with elevated PM_2.5_ concentrations likely creating heightened exposure scenarios. The analysis combined a number of data sets to show that compliance with a range of alternative annual and 24-hr PM_2.5_ standard groupings would affect a large fraction of the total population in the Northeast. This work finds that current PM_2.5_ standards in the eight-state study area affect only 16% of the general population, who live in counties that do not meet the existing annual/24-hr standard of 15/65 μg/m^3^. More protective PM_2.5_ standards recommended or enacted by California and Canada would protect 84–100% of the Northeast population. Standards falling within current ranges recommended by the U.S. EPA would protect 29–100% of the Northeast population. These considerations suggest that the size of general and susceptible populations affected by the stringency of alternative PM standards has broad implications for risk management and direct bearing on the U.S. EPA’s current NAAQS review and implementation.

Exposure to ambient fine particulate matter [particulate matter (PM) with an aerodynamic diameter ≤2.5 μm (PM_2.5_)] has been associated with a wide range of PM-related human health effects in general populations, including the aggravation of heart and lung disease and premature mortality (Brook et al. 2004; Holgate et al. 1999; Samet et al. 2000). The Clean Air Act of 1970 (CAA 1970) mandates the U.S. Environmental Protection Agency (EPA) to set health-based National Ambient Air Quality Standards (NAAQS) for certain pollutants known to be hazardous to human health, including PM. NAAQS provisions require the U.S. EPA to establish standards requisite to protect public health with an adequate margin of safety at a level that avoids unacceptable risks. Legislative history has interpreted the PM NAAQS margin of safety provision as requiring the protection of both general populations and sensitive subpopulations, or those subgroups potentially at increased risk for ambient particle health effects (National Air Quality Standards Act of 1970). Accordingly, the PM NAAQS—which are currently under review by the U.S. EPA—are intended to protect the health of the most sensitive members of society as well as the general population.

During the last decade, regulatory agencies have increasingly recognized that persons sensitive or susceptible to PM are more numerous and diverse than once thought. To achieve the public health protection called for by the CAA, the National Research Council (NRC) has recommended that subpopulations at increased risk from PM pollution should be identified and the nature and magnitude of their risk understood in the context of standard setting (NRC 2004). These groups comprise a large fraction of the U.S. population, including people with respiratory disease, heart disease, or diabetes; older people; young children; and populations experiencing heightened exposure levels (e.g., those engaged in outdoor work or exercise) [California Air Resources Board (CARB) 2002; U.S. EPA 2004a, 2004b].

Despite regulatory efforts over the past 40 years to improve air quality, the protection of public health with an adequate margin of safety is constrained by the inability of scientists to determine a safe level of exposure to PM_2.5_ below which populations are safe (Daniels et al. 2004; DiBattista and Brown 2003; Schwartz et al. 2002). The American Thoracic Society’s (ATS) statement on the nature of an adverse health effect of air pollution notes that although the NAAQS affords health protection to subgroups with increased susceptibility to air pollution using a margin of safety provision, this margin has not been quantified (ATS 2000). Given the likely heterogeneity of individual responses to air pollution, the severity of health effects experienced by a susceptible subgroup may be much greater than that experienced by the population at large (Zanobetti et al. 2000). Therefore, varying host susceptibility factors may hinder adequate protection of an entire population, even at low exposure levels [ATS 2000; Peters et al. 2004; World Health Organization (WHO) 2004].

Notwithstanding the limitations of current standard-setting methods, ambient air quality standards do ultimately determine the number of persons affected by air pollution (Deck et al. 2001). The more stringent the standard, the greater the emission reduction required and the more extensive the control strategies used to reduce PM concentrations. Reduction in ambient PM levels presumably reduces the public health toll exacted by PM pollution. However, given the current lack of an accepted threshold level for adverse health effects, any nonzero PM standard represents the air-pollution–related health burden that policy makers consider “acceptable” (Peters et al. 2004). This presents an important and challenging public health question because PM standards are the fulcrum on which society decides how many people will be at increased health risk to ambient PM. Furthermore, there may be variation in PM–health outcome associations for different subgroups and for different geographic regions, including the northeastern United States, which require consideration in the standard-setting process.

We assessed the extent to which compliance with various combinations of alternative PM_2.5_ standards would provide supplemental protection to general populations and susceptible subgroups in the northeastern United States. We first conducted a state-of-knowledge review of key regulatory and research organizations in the United States and Canada to determine which subgroups were considered to be at elevated risk to PM. We then integrated existing demographic and disease or health condition prevalence databases from the U.S. Census Bureau and Centers for Disease Control and Prevention (CDC) with various combinations of PM_2.5_ annual and 24-hr U.S. EPA design values generated from a network of air pollution monitoring sites across an eight-state Northeast study region. This analysis estimated the number of general population and susceptible subgroups in the northeastern United States that would benefit from compliance with alternative U.S. EPA annual and 24-hr mass-based PM_2.5_ standards. We believe the methodologic approach used provides an evaluative tool that may help decision makers place into regulatory context health data appropriate to the current review of the PM NAAQS. The analysis makes evident the public health implications of selecting among alternative PM_2.5_ standards with different degrees of health protection.

## Materials and Methods

We identified subpopulations considered potentially at elevated risk for adverse health effects related to PM by reviewing recent health assessment reviews and research reports. These included the Canadian Council of Ministers of the Environment’s (CCME) human health effects of PM_2.5_ report in support of the Canada-wide standards (CCME 2004); the CARB’s staff report to consider amendments to the ambient air quality standards for PM and sulfates (CARB 2002); the U.S. EPA’s PM criteria document (U.S. EPA 2004b), PM staff paper (U.S. EPA 2005), and Particulate Matter Research Program progress report (U.S. EPA 2004a); and comments provided by the NRC’s fourth report on research priorities for airborne PM (NRC 2004). To the extent that the four organizations identified or commented on subgroups likely or possibly at increased risk to PM, we estimated the magnitude of these subgroups for an eight-state study area (Connecticut, Maine, Massachusetts, New Hampshire, New Jersey, New York, Rhode Island, and Vermont) where data were sufficient. Common subgroups identified included susceptibility by age group, preexisting disease or health condition, heightened exposure, and socioeconomic status. Sufficient demographic and health prevalence data allowed for the estimation of subgroup size using age group and preexisting disease or health condition indicators. To a lesser extent, heightened exposure subgroups were also estimated using population density data.

We calculated age subgroup sizes from the 2000 Census (U.S. Census Bureau 2000) and matched preexisting disease or health condition indicators to available prevalence rates generated by recently published CDC health surveys desegregated by either state or Northeast region. Adult (≥ 18 years) self-reported asthma rates (ever) were obtained from the 2002 Behavioral Risk Factor Surveillance System (BRFSS), which was state specific. Lifetime asthma was defined as an affirmative response to the question “Have you ever been told by a doctor (nurse or other health professional) that you have asthma?” (CDC 2002a). We calculated the mean lifetime asthma prevalence rate for the eight states in the study area from each state-level prevalence rate. Adult sinusitis rates (preceding 12 months) and chronic bronchitis rates were obtained from the 2000 U.S. Adult National Health Interview Survey (NHIS) for the northeastern United States. The NHIS defines the northeastern United States as the six New England states, plus New Jersey, New York, and Pennsylvania. Respondents were asked in separate questions whether they had been told by a doctor or other health professional in the past 12 months that they had sinusitis or bronchitis (CDC 2003a).

We acquired adult cardiac prevalence rates from the 2000 NHIS for the northeastern United States (CDC 2003a). In separate questions, respondents were asked if they had ever been told by a doctor or other health professional that they had hypertension (or high blood pressure), coronary heart disease, angina (or angina pectoris), heart attack (or myocardial infarction), or any other heart condition or disease not already mentioned. Persons had to have been told on two or more different visits that they had hypertension, or high blood pressure, to be classified as hypertensive. Heart disease was defined to include coronary heart disease, angina pectoris, heart attack, or any other heart condition or disease (CDC 2003a). We obtained adult diabetes prevalence rates (ever) from the 2001 BRFSS report, which was state specific. Diabetes was defined as an affirmative response to the question “Have you ever been told by a doctor that you have diabetes?” (CDC 2002b).

We acquired child (< 18 years) respiratory allergies (preceding 12 months) and asthma (ever) prevalence rates from the 2001 U.S. Children NHIS for the northeastern United States (CDC 2003b). Allergy rates were based on the following questions: “During the past 12 months, has [child’s name] had any of the following conditions? Hay fever? Any kind of respiratory allergy?” Asthma rates were based on the question “Has a doctor or other health professional ever told you that [child’s name] has asthma?” (CDC 2003b).

To integrate demographic and health prevalence databases with various combinations of PM_2.5_ annual and 24-hr U.S. EPA design values generated from a network of air pollution monitoring sites, federal reference method (FRM) PM_2.5_ air pollution data from 2000, 2001, and 2002 were obtained from the U.S. EPA’s air quality system in August 2003 for 127 FRM monitors in U.S. EPA Region 1 (six New England states) and Region 2 (New Jersey, New York), 65 FRM monitors outside these regions in bordering states (Delaware, Maryland, and Pennsylvania, as well as the District of Columbia), and three Interagency Monitoring of Protected Visual Environments (IMPROVE) sites in Regions 1 and 2 [U.S. EPA 2003a; Visibility Information Exchange Web System (VIEWS) 2003]. Within the 2000–2002 period, 192 PM monitoring sites had data in all 12 quarters. Data flagged with the forest fire exemption for 2002 were removed. More than 75% of the 192 sites had better than 50% data capture within each quarter. Data completeness affecting the remaining sites was primarily isolated to one quarter. For sites with collocated monitors, the primary monitor at a site was used to determine the PM_2.5_ concentration (27 pairs of 192 monitors). Although less than half of the primary monitors satisfied the 75% data completeness criteria, no substitution from collocated monitors was attempted.

To determine whether data completeness would affect the relationship between the annual and 24-hr standards at each site, the 81 sites meeting the U.S. EPA’s strict 75% completeness requirement for 12 consecutive quarters were compared with 111 sites that did not meet completeness requirements. Regression equations and slopes between the two monitoring data sets were statistically indistinguishable. The regression (where *y* is the level of the 24-hr standard and *x* is the level of the annual standard) for the subset of monitors with complete data was *y* = 1.86*x* + 10.43 (*R* ^2^ = 0.76). The regression for the subset of monitors with incomplete data was *y* = 1.82*x* + 10.90 (*R*^2^ = 0.78). One data point was excluded from the linear regression because of its undue influence by virtue of its extreme value pair. Inclusion of this point changed the regression to *y* = 2.00*x* + 8.79 (although this slope is also statistically equivalent to that of the incomplete data).

To estimate the number of persons living in counties not likely to meet different combinations of alternative annual and 24-hr PM_2.5_ standards, 3-year average annual and 24-hr design values were calculated for all counties (150) in the eight-state study area and integrated with Census county-level population data using ArcGIS software (version 8.2; ESRI, Redlands, CA). Design values for state data were generated in adherence with the U.S. EPA’s criteria for determination of design values (U.S. EPA 1997, 1999). Alternative standard combinations were put forward for annual standards ranging from 11 to 15 μg/m^3^ (1-μg/m^3^ intervals) and for 24-hr (98th percentile) standards ranging from 20 to 65 μg/m^3^ (5-μg/m^3^ intervals). These ranges were selected to encompass recent California, U.S. EPA, and CCME recommended PM_2.5_ ranges or selected standards.

Design values for the 70 counties with monitors were assigned from the highest monitored levels in each county for 2000–2002. Design values for 80 counties lacking monitors were generated by interpolating county-level monitored design value data from 104 monitors within the eight-state study region and 61 monitors outside the region for border counties. An interpolation scheme was employed using inverse distance-squared weighting for the six nearest monitors within a 111-km radius (corresponding to 1° latitude). Massachusetts and New Hampshire had very few sites with complete data for the 3-year period, requiring an approximation of design values for counties in those states. For the other counties in the eight-state study region, the annual design values used were generally within 0.2 μg/m^3^ of those reported by the U.S. EPA using customary guidelines for data substitution and completeness determinations (U.S. EPA 2003b).

We calculated the number of susceptible persons identified as potentially at elevated risk to PM living in counties with PM_2.5_ levels exceeding various annual/24-hr standard combinations for age subgroups and persons with preexisting health conditions using Census age demographic and BRFSS and NHIS health survey prevalence data (CDC 2002a, 2002b, 2003a, 2003b; U.S. Census Bureau 2000). Prevalence rates were multiplied by the number of persons in respective adult and child age groups estimated to be living in counties with PM_2.5_ levels exceeding PM_2.5_ standard combinations.

Differing forms of PM_2.5_ annual and 24-hr primary standards of selected U.S. and Canadian government agencies were normalized to facilitate general comparisons across agencies. This allows for the estimation of how other agency’s standard levels correspond to the U.S. EPA’s standard level. Relationships were generated using 2000–2002 data from 192 PM monitors located in the eight states and border states of the study region. To compare California’s 1-year not-to-be-exceeded (NTBE) target annual standard with the U.S. EPA’s 3-year mean annual standard, the relationship between the 3-year annual average and the individual annual averages from the 3 years was reviewed. The highest 3-year average annual value for which no individual year exceeded the California standard was 11.5 μg/m^3^. However, several sites showed a 3-year average lower than this where an individual year had exceeded 12 μg/m^3^. There were no annual excursions above the 12 μg/m^3^ level for a site when the 3-year annual average was < 11.0 μg/m^3^. These values (11.0–11.5 μg/m^3^) represent a reasonable range of equivalency between a 3-year annual average and a 1-year annual average NTBE standard form.

The relationship between California’s proposed 1-year NTBE target 24-hr standard and the U.S. EPA’s 3-year mean 98th percentile 24-hr standard was also derived from the 3-year data set (U.S. EPA 2003a; VIEWS 2003). Unlike the annual standard, California’s 24-hr standard is structured to allow the exclusion of one extreme day per year over 3 years. To account for these potential extreme day exclusions, the 24-hr values were ranked over 3 years and exclusions were permitted based on total available collected samples; for each 365 sample days, the highest concentration value was excluded. For most sites that sampled on a 1-in-3-day schedule, no exclusions were allowed. For 24-hr sampling sites, generally the top 2 concentration days were excluded, leaving the third highest day as the 24-hr standard level. Because the lowest maximum 24-hr value for any site was > 25 μg/m^3^, a conservative corresponding 98th percentile form value (18 μg/m^3^) was extrapolated from the linear regression between the maximum value at a site (after exclusion) over 3 years and the 3-year average 98th percentile value. A second approach relied on the regression relationship of the 3-year average of the year-specific maximum values and the 3-year average 98th percentile, yielding 20 μg/m^3^. This approach is roughly equivalent to excluding 1 extreme day over 3 years. These values were used to establish the tabulated 98th percentile range of 18–20 μg/m^3^ that corresponds to the 25-μg/m^3^ 24-hr maximum.

## Results

We conducted a review of recent PM reports from CARB, the U.S. EPA, CCME, and NRC to assess whether ambient PM is believed to have a disproportionate effect or increased risk on certain populations. This was accomplished by comparing how the various organizations conceived of sensitive populations and defined determinants of sensitivity among subgroups. Previous research on sensitivity or susceptibility has noted varying conceptual approaches to defining the terms and subgroups, given different interpretations of the state of knowledge (ATS 2000; ATS Committee 1996; Parkin and Balbus 2000; Pope 2000). The ATS has broadly defined “susceptibility” as including extrinsic factors, such as the profile of exposure to other pollutants, and intrinsic factors, such as genotype. As scientific advances more precisely identify those at risk within the distribution of the degree of susceptibility, it may become increasingly challenging to regulate outdoor air pollution to assure protection for all individuals against adverse health effects. Such effects may already or eventually include biomarker changes, health-related quality of life, physiologic impact, symptoms, clinical outcomes, and mortality (ATS 2000).

The U.S. EPA and NRC each provided definitions of susceptibility and construed the term differently. The U.S. EPA’s PM criteria document defined susceptibility as generally encompassing “innate or acquired factors that make individuals more likely to experience effects with exposure to pollutants” (U.S. EPA 2004b). Innate susceptibility can entail genetic or developmental factors, whereas acquired susceptibility may result from age, disease, or personal risk factors such as smoking, diet, or exercise. The U.S. EPA also referred to the concept of increased vulnerability to pollution-related effects, as distinct from susceptibility, because of factors including socioeconomic status or experiencing “particularly elevated exposure levels” (U.S. EPA 2004b). NRC’s Committee on Research Priorities for Airborne Particulate Matter was charged to gauge research progress on susceptible subpopulations by evaluating new evidence that has appeared since 1998. NRC commented on a broadening scope of health concerns, including an increasing number of adverse health outcomes associated with PM and related susceptible subpopulations. The committee referred to groups as “particularly susceptible” to the effects of air pollution based on one or more of the following factors: *a*) increased exposure due to longer-duration and/or higher-than-normal pollution concentrations, *b*) higher delivered dose due to physiologic factors, and *c*) a greater health response than the general population to a given dose of air pollution (NRC 2004).

Overall, the current list of subgroups for which PM likely or possibly has disproportionate health effects is reasonably congruent across the four organizations. Six categories or determinates of susceptibility were identified: age, preexisting disease, heightened exposure, genetic makeup, sex, and socioeconomic status. The level of scientific understanding associated with research findings for these categories was characterized by groups to which exposure to PM likely or possibly has disproportionate health effects and groups to which exposure to PM is of concern, but overall evidence is insufficient or limited.

Two categories listed as likely or possibly affected by PM were identified explicitly in all four reports. These categories comprised population subgroups defined by age (infants, children, and persons ≥ 65 years of age) and by preexisting disease (cardiopulmonary disease and diabetes). The category defined by heightened exposure levels (e.g., populations involved in outdoor exercise, outdoor work, and living near high PM sources) was either listed as likely or possibly affected by PM or was not considered explicitly.

The NRC and U.S. EPA identified population subgroups defined by heightened exposure levels as likely or possibly affected by PM in report sections devoted specifically to assessing susceptible or vulnerable subpopulations (NRC 2004; U.S. EPA 2004b). However, both the U.S. EPA and NRC offered different interpretations of whether these groups are “susceptible” or “vulnerable.” The NRC defined groups with heightened exposure status—such as proximity to source or outdoor exercise—as susceptible, whereas the U.S. EPA defined these groups as vulnerable. CARB and CCME reports recognized the potential impact of heightened exposures on subpopulations, but not within sections specifically devoted to susceptible or vulnerable populations (CARB 2002; CCME 2004). Heightened exposure as a determinate of increased risk was instead discussed in other sections (e.g., human exposure assessment) or by reference to scientific investigations in sections devoted to epidemiologic field studies.

The U.S. EPA characterized socioeconomic status as both likely and possibly having disproportionate health effects and of concern, but with insufficient or limited overall evidence (U.S. EPA 2004b). This divergence of outcomes relates to long-term epidemiologic studies that find PM–mortality risk may be greater for those with lower socioeconomic status, whereas time-series epidemiologic studies provide less evidence of effect modification for short-term exposure effects by socioeconomic status.

Finally, four categories were either not considered in all the research reports or, if listed, were believed to be of concern but with insufficient evidence. These subgroup categories were defined by age (fetus), genetic makeup, sex, and socioeconomic status (for time-series studies).

Based on the framework of susceptibility criteria established in the review, age, preexisting disease, heightened exposure, and socioeconomic categories were identified as likely or possibly at increased risk to PM. In the eight-state northeastern U.S. study area, data were analyzed to estimate the magnitude of susceptible groups in the age and preexisting disease categories, and to a lesser extent to estimate the heightened exposure category. Tables 1 and 2 illustrate that subgroups susceptible to PM represent a large fraction of the northeastern U.S. population. Table 1 shows the population age group distributions for the eight-state study region. The number and percentage of persons in age-related susceptible subgroups are indicated for < 3-year, 3- to 17-year, and ≥65-year age classes. Thirty-eight percent or 15.6 million persons of the region’s total population (41.3 million persons) were infants, children, or older adults.

Table 2 summarizes information on the prevalence of chronic cardiopulmonary conditions and diabetes in the northeastern U.S. population. The number of adults (≥ 18 years of age) and children (< 18 years of age) in the northeastern United States with cardiac and respiratory conditions and diabetes was estimated by compiling recent BRFSS and NHIS surveys on disease or health condition prevalence between 2000 and 2002 (CDC 2002a, 2002b, 2003a, 2003b). Adults with preexisting heart and lung conditions ranged from approximately 4 to 18% of the total northeastern adult population. For respiratory conditions, 15% have been told by a doctor or other health professional they have sinusitis (preceding 12 months), 13% asthma (ever), and 4% chronic bronchitis (preceding 12 months). For circulatory conditions, 10% of the adult population has received a diagnosis of heart disease (ever) and 18% hypertension (ever). The percentage of adults with hypertension was likely > 18% because persons may have a silent or undiagnosed condition. The CDC’s National Health and Nutrition Examination Survey found that measured hypertension (physical examination) in the United States among persons ≥ 20 years of age is 30% (National Center for Health Statistics 2003). Six percent of adults in the northeastern United States have ever been told by a doctor they have diabetes. Twelve percent of children have been diagnosed with respiratory allergies (preceding 12 months). Fifteen percent of children have been diagnosed with asthma at some point in their life. Comparing across age groups, cardiovascular conditions were more common among older age groups, whereas asthma prevalence was higher in children.

**Figure 1:**
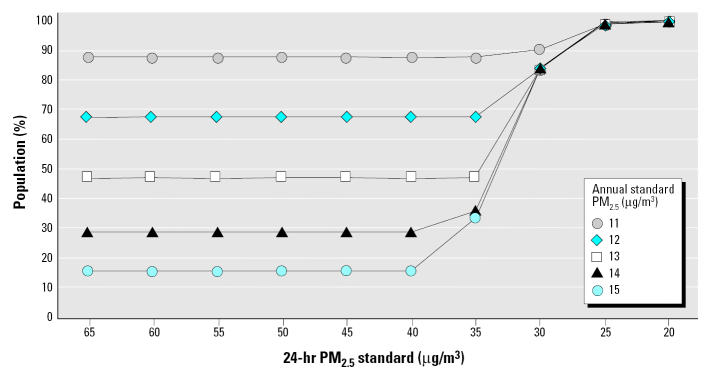
Percentage of the northeastern population that would benefit from compliance with alternative annual/24-hr PM_2.5_ (98th percentile) standards.

**Figure 2:**
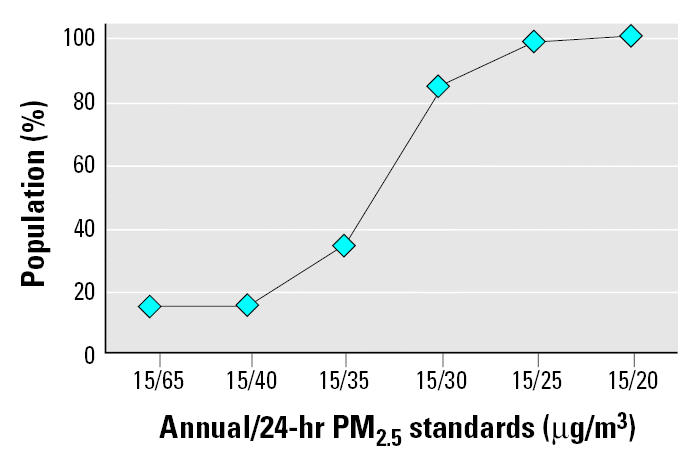
Percentage of northeastern susceptible age subgroups that would especially benefit from compliance with alternative annual/24-hr PM_2.5_ (98th percentile) standards.

**Figure 3:**
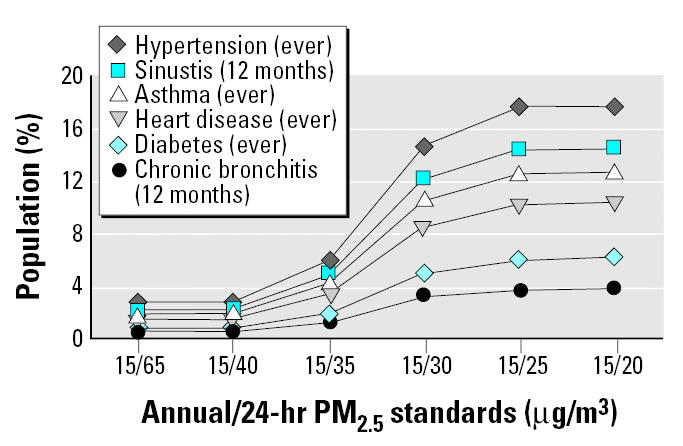
Percentage of all adults that would especially benefit (members of subgroups with preexisting health conditions) from compliance with alternative annual/24-hr PM_2.5_ (98th percentile) standards.

**Figure 4:**
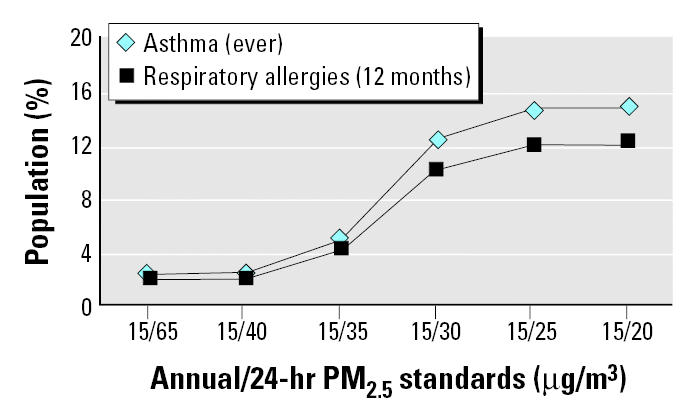
Percentage of all children that would especially benefit (members of subgroups with pre-existing health conditions) from compliance with alternative annual/24-hr PM_2.5_ (98th percentile) standards.

Given the need to identify the nature and magnitude of susceptible population risk in the context of standard setting (NRC 2004), compliance with various combinations of alternative PM standards could benefit general populations and especially benefit susceptible populations in the northeastern United States. Figures 1–4 reflect the benefits from improved air quality as a result of additional PM_2.5_ control strategies.

Figure 1 shows the percentage of the eight-state total population living in U.S. EPA Regions 1 and 2 counties with PM_2.5_ concentrations less or greater than various combinations of annual and 24-hr (98th percentile) alternative standards and levels for 2000–2002. The U.S. EPA’s current annual and 24-hr PM_2.5_ standards are 15 and 65 μg/m^3^ (98th percentile), respectively. As indicated in Figure 1, 16% of the region’s population currently lives in counties that do not meet the existing annual/24-hr standard of 15/65 μg/m^3^. Were the revised annual standard of 15 μg/m^3^ to remain unchanged, the percentage of the total population living in counties not meeting annual/24-hr standards would change only after the 24-hr standard is lowered to < 40 μg/m^3^. A 24-hr standard of 30 μg/m^3^ coupled with an annual standard of 12, 13, 14, or 15 μg/m^3^ would result in 84% of the population living in counties that would not meet the regulation. As depicted in Figure 1, compliance with alternative annual/24-hr standard setting in U.S. EPA Regions 1 and 2 would benefit populations if the annual standard moved to < 15 μg/m^3^ or the 24-hr standard moved to < 40 μg/m^3^. An annual standard of 12 μg/m^3^ would result in 68% of the population living in counties that would not meet the regulation, whereas a 24-hr standard of 20 μg/m^3^ would result in 100% of the population living in counties not meeting the regulation.

Figures 2–4 condense the analysis to combinations of an annual standard of 15 μg/m^3^ with alternative 24-hr standards ranging from 65 down to 20 μg/m^3^ (98th percentile). The condensed annual/24-hr range of alternatives captures the entire sphere of all annual 11–15 μg/m^3^/24-hr 20–65 μg/m^3^ ranges with respect to affected populations. As presented in Table 1, 38% of the eight-state region’s population is composed of infant, children, and older adult subgroups considered susceptible to PM. Figure 2 shows the percentage of these subgroups living in counties with PM_2.5_ concentrations less or greater than various combinations of annual and 24-hr (98th percentile) alternative standards and levels for 2000–2002. In Figure 2, the current annual/24-hr standard of 15/65 μg/m^3^ results in 15% of the region’s susceptible age groups living in counties with PM_2.5_ levels at or above the standard. Compliance with a revised annual/24-hr PM_2.5_ standard of 15/30 μg/m^3^ would especially benefit 84% of the region’s susceptible age groups with improved air quality.

Figures 3 and 4 show adult and children subgroups with preexisting health conditions considered to be determinates of susceptibility, by ages ≥ 18 years and < 18, respectively, as a percentage of the total population. These sub-groups live in counties with PM_2.5_ concentrations less or greater than various combinations of annual and 24-hr (98th percentile) alternative standards and levels for 2000–2002. In Figure 3, adult populations with preexisting health conditions contributing to susceptibility represent 0.6–3% of the total adult population living in counties with PM_2.5_ levels above the current annual/24-hr standard of 15/65 μg/m^3^. A revised annual/24-hr PM_2.5_ standard of 15/20 μg/m^3^ would especially benefit about 4–18% of the total population, or 100% of all adults in the northeastern region currently estimated to have these health conditions. In Figure 4, child populations with preexisting respiratory conditions represent 2–2.4% of the total children population living in counties with PM_2.5_ levels above the current annual/24-hr standard of 15/65 μg/m^3^. A revised annual/24-hr PM_2.5_ standard of 15/20 would especially benefit about 12–15% of the total population, or 100% of all children in the northeastern region currently estimated to have these health conditions.

In addition to age and preexisting disease or health condition indicators, heightened air pollution exposure status represents another category of susceptibility wherein populations are possibly or likely at increased risk to PM. Possible subpopulations affected include outdoor workers, children and adults physically active outdoors, and people living near high-intensity sources. Presently, there is no universal indicator used to quantify the number of persons that may be at risk because of heightened exposure status. Given that combustion-source particulate air pollution is common to many urban environments, these areas may function as examples of environments in which populations commonly experience heightened PM levels. Urban airsheds in the northeastern United States experience elevated 24-hr average and annual mean PM concentrations and are home to numerous intense sources [Cass et al. 1999; NARSTO (formerly North American Research Strategy for Tropospheric Ozone) 2004].

Using population density as an indicator of an urban-scale demographic, 2000 U.S. Census data are presented in Table 3. The northeastern region’s urban areas, defined as having census tract population densities greater than 1,000 persons/miles^2^, consisted of 6% of the total land mass and were home to about 30 million persons or 72% of the region’s total population of 41.3 million persons. The percentage of child, adult, and elderly age subgroups living in urban areas was nearly identical, ranging from 71 to 73% across groups, and comprised 27% of the region’s total population. The density of this eight-state region is among the highest in the nation, because five of eight states (New Jersey, Rhode Island, Massachusetts, Connecticut, New York) are among the six most densely populated states in the United States. Thus, most persons—across child, adult, and elderly age groups—in the northeastern United States live in densely populated urban areas that are also characterized by elevated PM levels and heightened exposure scenarios.

## Discussion

This study draws attention to public health issues facing regulators charged to minimize the harmful impact of ambient PM_2.5_ on populations. Our analysis of northeastern U.S. monitoring and demographic data suggests the population size of susceptible groups—a key indicator of the potential impact of PM_2.5_ exposure on public health—is extensive. Although additional knowledge is needed about the biologic mechanisms and host characteristics involved in susceptibility, a variety of groups are likely more susceptible or vulnerable to PM. Within the eight-state study area, 38% of the total population are < 18 or ≥ 65 years of age, 4–18% of adults have cardiopulmonary or diabetes health conditions, 12–15% of children have respiratory allergies or lifetime asthma, and 72% of all persons (across child, adult, and elderly age groups) live in densely populated urban areas with elevated PM_2.5_ concentrations likely creating heightened exposure scenarios. In addition, current PM_2.5_ standards in the eight-state study area affect only 16% of the general population, who live in counties that do not meet the existing annual/24-hr standard of 15/65 μg/m^3^. A combination of more stringent annual/24-hr standards would result in a larger percentage of the population living in counties that would not meet the regulation; these populations would therefore benefit from greater emission reduction requirements and more extensive control strategies to reduce PM concentrations.

When taking into account susceptible subgroups, it is difficult to set standards consistent with the intent of the CAA—which stipulates that the U.S. EPA establish primary NAAQS at a level that protects sensitive populations—because of science’s inability to confirm the existence of a PM_2.5_ threshold level under which there are no health effects. In response, major regulatory organizations in the United States and Canada set enforceable or target standard levels to limit PM_2.5_ concentrations below those where epidemiologic evidence is most consistent and coherent. This approach recognizes both the strengths and the limitations of the full range of scientific and technical information on the health effects of PM, as well as associated uncertainties.

The interpretation of available data by different standard-setting bodies may reflect the varying levels of health protection required by the controlling statute and the level of public health protection commitment. Table 4 estimates the relationship among current or recently recommended California, Canada, and U.S. PM_2.5_ standards by normalizing differing annual and 24-hr forms. This facilitates a comparison of corresponding standard levels and forms that differ among the three agencies. Both Canada and the U.S. EPA currently use a 98th percentile 3-year average form for the 24-hr PM_2.5_ standard. Canada’s 24-hr standard of 30 μg/m^3^ would result in 84% of the eight-state Northeast study area population living in counties that would not meet the regulation. Although Canada does not have an annual standard, the U.S. EPA’s annual PM_2.5_ standard form is expressed as the annual arithmetic mean averaged over 3 years.

California’s proposed (later deferred) 24-hr and adopted annual standard form are based on year-to-year NTBE values, which include maximum monitoring values and are more stringent than 3-year and 98th percentile forms. Were California’s proposed 24-hr standard of 25 μg/m^3^ (NTBE) converted into a 98th percentile form, the standard would range from 18 to 20 μg/m^3^. This 24-hr standard would result in 100% of the eight-state Northeast study area population living in counties that would not meet the regulation. Were California’s adopted annual standard of 12 μg/m^3^ (NTBE) converted into the U.S. EPA’s form, the standard would range from 11 to 11.5 μg/m^3^. An annual standard of 11 μg/m^3^ would result in 88% of the eight-state Northeast study area population living in counties that would not meet the regulation.

Although differences in health-related PM air pollution standard setting are common across agencies (Benner 2004), PM_2.5_ exposure associations with adverse health effects may well extend to levels lower than the most stringent recommended target standards. Even if PM_2.5_ NAAQS attainment were reached, health risks within the U.S. population would not be totally eliminated. As demonstrated by this study, however, the stringency of PM_2.5_ standards can determine the magnitude of the PM_2.5_-related health burden that decision makers choose to place on the population. Within the framework of standard-setting logic, incrementally more stringent standards would offer the expectation of increased public health protection from PM_2.5_ exposures. Epidemiologic evidence shows that large-scale interventions and natural reductions in ambient PM have resulted in decreases in disease and death (Clancy et al. 2002; Laden et al. 2001; Pope 1991). This underscores the importance of setting appropriately stringent PM_2.5_ standards to trigger control measures intended to reduce ambient PM_2.5_.

A central limitation of the study was its inability to generate additive estimates of total susceptibility across the eight-state study region. The population as a whole is considered diverse in its susceptibility to inhaled pollutants, and persons may be represented in multiple categories of susceptibility. The range of sensitivity among persons is uncertain because variations in PM exposure, PM dose, and host-related factors can cause exposed people to be more susceptible.

The study could have benefited from more refined estimates of factors determining susceptibility in urban populations, including those experiencing heightened exposures such as outdoor worker, child, athlete, other exercising adult and child, and commuter subgroups. The study also did not account for other potential susceptibility indicators, such as socioeconomic status, which may influence exposure scenarios and health disparities, especially among urban populations (American Lung Association 2001). Moreover, a consideration of projected demographic shift and epidemiologic transitions likely would have augmented the import of study findings. For example, in the U.S. populations ≥65 years of age are projected to increase from 12.4% in 2000 to 19.6% in 2030, or from about 35 million to 71 million, respectively. Approximately 80% of all persons in this age cohort have at least one chronic condition, 50% have at least two, and overall chronic diseases such as diabetes and heart disease affect older adults disproportionately (Anderson and Smith 2003; Goulding et al. 2003).

In addition, the study did not quantify the potential for a varying profile of susceptibility to PM across spatial scales. The NHIS study findings were regional and included the eight-state study area and Pennsylvania (CDC 2003a, 2003b). The BRFSS asthma and diabetes surveys provided prevalence rates by state, but only for adults (CDC 2002a, 2002b). Regional and state resolution scales do not enable one to distinguish prevalence rates between, for example, urban and non-urban populations with respect to specific states or other geographic scales.

Concerning the integration of prevalence rate data with design value estimates, the uniform application of CDC prevalence rate data to populations living in counties not meeting alternative PM_2.5_ standards assumes that CDC data for the region are representative of those counties. With respect to the study’s use of monitoring data, the assessment followed U.S. EPA methods by assigning the highest annual or 24-hr design values as the design values for the entire county (U.S. EPA 1999). Likewise, for those counties without monitors, the highest annual or 24-hr interpolated levels were used from counties with monitors. This method could overestimate the number of persons exposed to PM_2.5_ concentrations at the county level. However, the study applied county-level population estimates to achieve greater resolution and accuracy. The U.S. EPA currently defines attainment/nonattainment areas by consolidated metropolitan statistical areas that aggregate counties (Holmstead 2003). Finally, application of a 3-year data set (2000–2002) incorporating a wide range of monitoring sites and concentration values allowed us to establish the relationship between various PM_2.5_ standard metrics. The inclusion of additional years to the analysis probably would not materially change this relationship unless factors driving PM concentrations across the northeastern region were suddenly to change. Since 2002, this has not happened.

The above limitations recommend more definitive data collection efforts, as future research using this study’s integrative analytical approach would benefit from improved knowledge about susceptible subpopulations and the use of highly spatially resolved monitoring data. This might be fostered by the U.S. EPA and U.S. Department of Health and Human Services cross-agency research platforms guiding future investigations, and further broadening of problem definitions in each organization. For example, the CDC and U.S. EPA might develop a common health survey framework to *a*) augment our understanding of specific subpopulations by exploring disease, vital, and behavioral variability among regions (or even states or metropolitan areas) across all age groups; *b*) provide information about urban-scale (and other scales, e.g., rural) health impacts—rather than gross national or regional-scale impacts; *c*) help explain putative heterogeneity of health effects in urban areas across U.S. regions as reported by epidemiologic studies; and *d*) gain insight into populations at high risk residing near source-dominated environments. These suggested approaches would provide policy makers with a greater understanding of how the U.S. EPA’s PM NAAQS recommendation will affect public health.

In conclusion, this study was conducted to assess the public health implications of the current PM NAAQS revision process. Using susceptibility criteria compiled from major regulatory and research reports, we found that a significant percentage of the eight-state region’s population is potentially susceptible to PM_2.5_, including 38% of the total population by age group and 4–18% of adults and 12–15% of children by preexisting health condition. More than 70% of the child, adult, and elderly population age groups in the study area live in urban areas that experience elevated PM_2.5_ concentrations and heightened exposure scenarios. This finding may be relevant to studies suggesting the potential for heterogeneity in U.S. city-specific excess risk estimates for acute health effects, including higher mortality coefficients in the Northeast (Dominici et al. 2002). We also devised an evaluative method that uniformly applied CDC prevalence rates for selected health conditions and Census age distributions to the number of persons living in areas with PM_2.5_ concentrations above annual/24-hr standard combinations. We found that currently only 16% of the eight-state region’s general population lives in counties that do not meet the annual/24-hr PM_2.5_ standards. However, a large fraction of the region’s total population would benefit and a large number of adult and children populations with chronic health conditions would especially benefit from compliance with PM_2.5_ levels less or greater than various combinations of annual and 24-hr average (98th percentile) concentrations currently under review by the U.S. EPA. More protective PM_2.5_ standards falling within ranges recommended by California and Canada would protect 84–100% of the general population.

## Figures and Tables

**Table 1:** Number and percentage of age subgroups living in the northeastern United States.

Age group (years)	No.	Percent
< 3	1,574,903	4
3–17	8,550,659	21
≥ 65	5,453,117	13
Total (< 18, ≥ 65)	15,578,679	38
18–64	25,734,645	62
Total (all ages)	41,313,324	100

**Table 2:** Prevalence and number of children and adults with specific preexisting disease conditions living in the northeastern United States.

Age group and health condition	Prevalence rate (%)	No.
< 18 years		10,125,562
Respiratory allergies (preceding 12 months)	12.2	1,235,319
Asthma (ever)	14.8	1,498,583
≥ 18 years		31,187,762
Sinusitis (preceding 12 months)	14.7	4,584,601
Asthma (ever)	12.8	3,992,034
Chronic bronchitis (preceding 12 months)	3.9	1,216,323
Hypertension (ever)	17.9	5,582,609
Heart disease (ever)	10.4	3,243,527
Diabetes (ever)	6.2	1,933,641

**Table 3:** Distribution of population age groups by nonurban and urban population density scales (persons/mi^2^ land area) in the northeastern United States.

	0–1,000 (94% of total land mass)		> 1,000 (6% of total land mass)		
Age (years)	No.	Percent total	No.	Percent total	Percent age group
< 18	2,915,526	7	7,210,036	17	71
18–64	7,008,390	17	18,726,255	45	73
≥ 65	1,460,005	4	3,993,112	10	71
Total	11,383,921	28	29,929,403	72	72

**Table 4:** PM_2.5_ primary standards of selected government agencies.

	California			U.S. EPA	
	2003, target^a^	2002, deferred^b^	Canada 2000, target^c^	1997, final	2005, recommended range^d^
24-hr standard					
Level (μg/m^3^)	NA	25	30	65	25–40
Form		NTBE of 98^th^ percentile	3-year average of 98th percentile	3-year average of 98th percentile	3-year average of 98th or 99th percentile
Normalized		~18–20	30	65	25–40
Annual standard					
Level (μg/m^3^)	12		NA	15	12–15
Form	NTBE			3-year average	3-year average
Normalized	~11–11.5			15	12–15

NA, not applicable.

a California’s new state standards amount to new clean air goals for the state and took effect in June 2003 (CARB 2002).

b California proposed a new 24-hr average standard for PM2.5 at 25 μg/m^3^, NTBE, in May 2002 but subsequently deferred a final decision (CARB 2002).

c Target implementation to be achieved by 2010 and ratified by ministers on June 2000.

d U.S. EPA (2005).
